# A stone in the bone

**DOI:** 10.1002/jmd2.12246

**Published:** 2021-10-08

**Authors:** Matthieu Halfon, Pierre Cochat, Sebastien Kissling, Nicolas Dattner, Laurence de Leval, Fadi Fakhouri, Menno Pruijm, Olivier Bonny

**Affiliations:** ^1^ Service of Nephrology Lausanne University Hospital Lausanne Switzerland; ^2^ Centre de référence des maladies rénales rares, Hospices Civils de Lyon Lyon France; ^3^ Department of Laboratory Medicine and Pathology Institute of Pathology, Lausanne University Hospital and University of Lausanne Lausanne Switzerland; ^4^ Department of Biomedical Sciences University of Lausanne Lausanne Switzerland

**Keywords:** bone, chronic kidney disease, hypercalcemia, oxalate, oxalosis, primary hyperoxaluria

## Abstract

Primary hyperoxaluria (PH) is a group of diseases due to mutations in genes coding for enzymes involved in oxalate metabolism. Three types of PH are identified depending on the gene mutated. Type 1 is the most frequent with 80% of the cases, while PH2 and PH3 are rarer. The severity of renal involvement varies between the three types. Indeed, between 60% and 80% of PH1 but only 20% of PH2 patients will reach end‐stage kidney disease. In PH3 patients, dialysis is uncommon. Because oxalate clearance is impaired in CKD patients, oxalate can precipitate in various organs leading to systemic oxalosis. We report an uncommon presentation of bone oxalosis associated with hypercalcemia in a dialyzed patient. This report emphasizes the difficulties to diagnose primary hyperoxaluria and the challenge of treating dialyzed patients.

## 1 CASE

**FIGURE 1:**
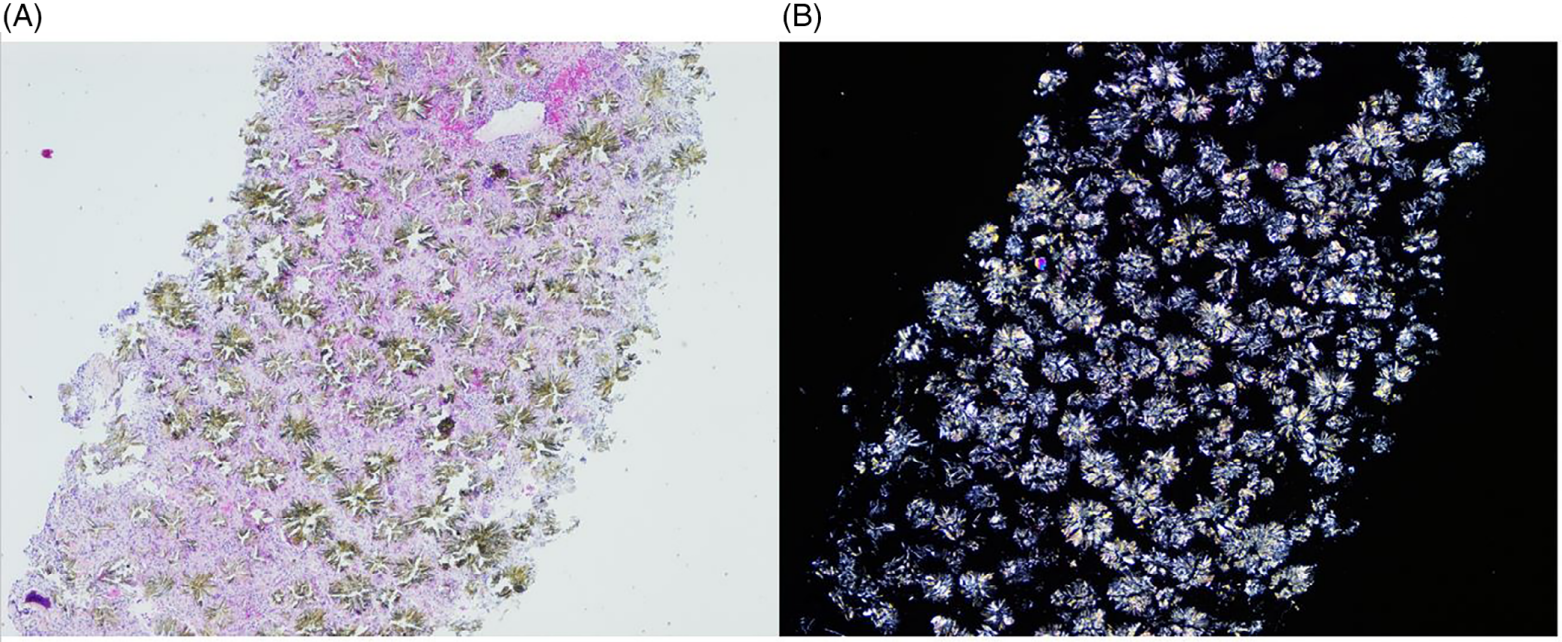
Optic microscopy of bone biopsy (HE staining). (A) 40× magnification. (B) Same area, 40× with polarized light

**FIGURE 2:**
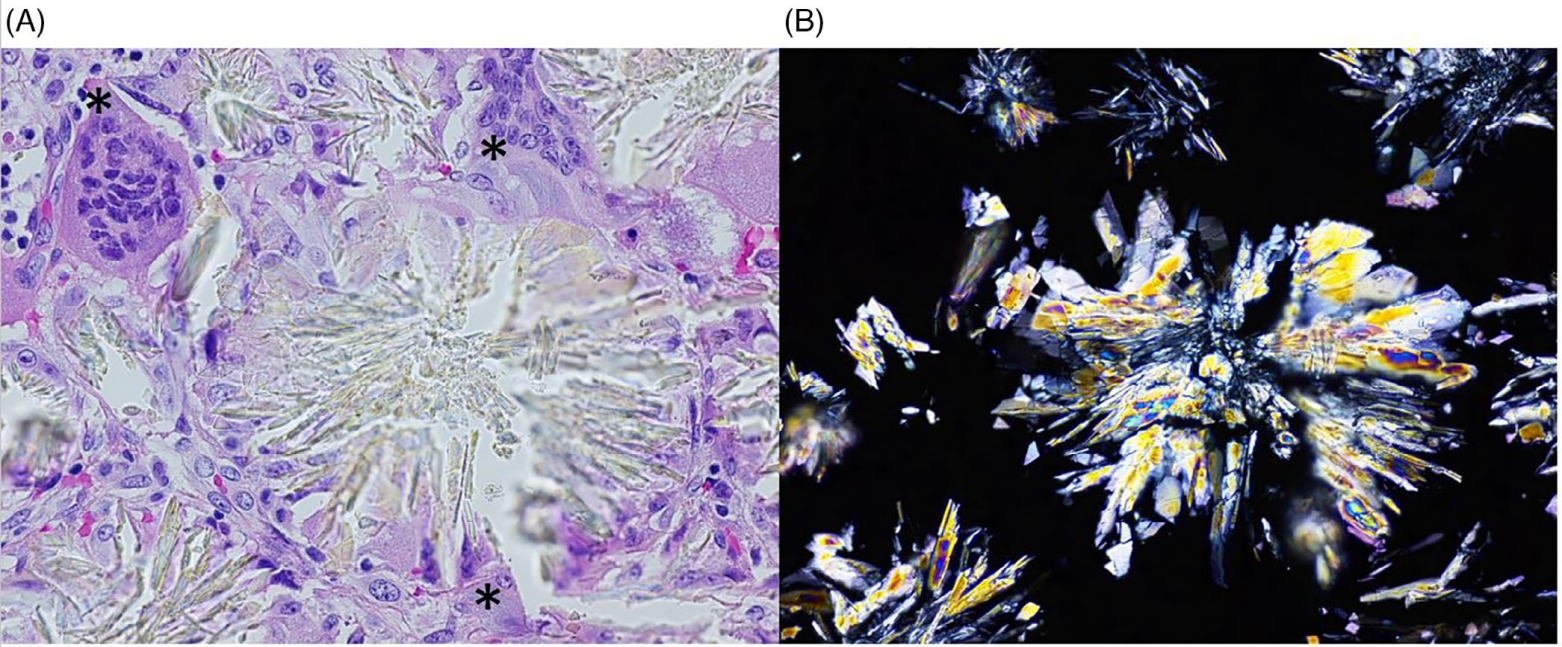
Optic microscopy of bone biopsy (HE staining). (A) 400× magnification. Note the presence of a giant cell (*). (B) Same area, 400× with polarized light

A 55‐year‐old female on chronic hemodialysis (HD) for 7 years presented with hypercalcemia at a routine monthly laboratory exam. End‐stage kidney disease (ESKD) had been attributed to recurrent episodes of pyelonephritis. Thirteen years before, she had experienced a renal colic and a computed tomography scan had shown bilateral kidney stones. She then underwent J‐J tube placement and extracorporeal shock‐wave lithotripsy. Analysis of the composition of the calculus revealed calcium oxalate monohydrate 100%. Eight months prior to the discovery of hypercalcemia, vitamin D supplements had been introduced for 25‐OH vitamin D deficiency. Eventually, the dose of post‐dialysis multivitamin complex, including vitamin C, was doubled 3 months before the occurrence of hypercalcemia in the context of the COVID‐19 pandemic. As the patient lost weight and was complaining of bone pain, a PET‐scan was performed showing multiple hypercaptations in the bones. A bone biopsy was performed showing multiple birefringent crystal deposits, surrounded by inflammatory infiltrates and granulomas (Figures 1 and 2). Infrared microscopy identified calcium oxalate monohydrated crystals, highly suspicious of primary hyperoxaluria (PH). Genetic analysis showed two pathogenic heterozygous variants of the *AGXT* gene (c.508G>T; p.Gly170Arg and c.106C>T; p.Arg36Cys) and a minor allele of the *AGXT* gene (c.32C>T; p.Pro11Leu) in the homozygous state confirming the diagnosis of PH type 1 (PH1).

PH is a group of rare, autosomal recessive diseases due to mutations in genes coding for enzymes involved in the oxalate metabolism. Patients usually present with a history of recurrent kidney stones and progressive CKD chronic kidney disease. As oxalate is mainly excreted by the kidneys, progression of the renal disease may result in impaired oxalate excretion and in a rise in serum oxalate that favors oxalate deposits in all tissues, leading to systemic oxalosis.^1^ The patient presented here suffered from bone oxalosis. Clinically, most patients with bone oxalosis suffered from bone pain, pathological fractures, or bone deformity.^2^ Studies have shown that bones of patients suffering from oxalosis present a decrease concentration of carbonate. This finding might explain the increased risk of fractures.^2^ Of note, measurement of bone mineral density by quantitative tomography correlates with oxalemia and disease severity.^3^, ^4^

Calcium oxalate deposits can also be observed in the heart, vessels, or retina, resulting in a wide clinical spectrum, and making the diagnosis often challenging.^1^ Indeed, between 20% and 52% of adult patients have developed ESKD at the time of diagnosis and almost 10% are diagnosed only after recurrence of the disease in the renal graft.^5^, ^6^ The treatment of PH1 patients with ESKD is challenging, as even intensive HD is not sufficient to balance oxalate production. Combined liver–kidney transplantation is the treatment of choice in CKD5/5D.^1^ Lumasiran is currently approved in patients with PH1 and holds promise for HD patients.^7^

## 2 SUPPLEMENTAL DATA: EXPLANATION OF HYPERCALCEMIA

Hypercalcemia is not a common finding in the course of PH and was never observed before in this patient. Hypercalcemia occurred shortly after the increase in the dose of vitamin D and of vitamin C‐containing multivitamins. Since vitamin C is a precursor of oxalate, we hypothesize that vitamin C supplements increased oxalate production and subsequently accelerated oxalate deposition in bones and granuloma formation (Figure 2B). Due to their 1‐alpha‐hydroxylase activity, granulomas may have transformed 25‐OH‐D to 1,25‐(OH)_2_‐D, thus stimulating intestinal calcium absorption, which led to hypercalcemia.^8^

## CONFLICT OF INTEREST

The authors report no conflict of interest.

## AUTHOR CONTRIBUTIONS

Matthieu Halfon, Menno Pruijm, and Olivier Bonny were the nephrologists in charge of the patient and wrote the manuscript. Pierre Cochat provided the analysis and the interpretation of the oxalemia and contributed to the manuscript. Sebastien Kissling and Fadi Fakhouri were in charge of the patient and contributed to the manuscript. Nicolas Dattner and Laurence de Leval were the pathologists who analyzed the biopsy.

## INFORMED CONSENT

A signed inform consent was obtain from the patient.

## References

[1] Cochat P , Rumsby G . Primary hyperoxaluria. N Engl J Med. 2013;369(7):649‐658. 23944302 10.1056/NEJMra1301564

[2] Bacchetta J , Farlay D , Abelin‐Genevois K , Lebourg L , Cochat P , Boivin G . Bone impairment in oxalosis: an ultrastructural bone analysis. Bone. 2015;81:161‐167. 26164477 10.1016/j.bone.2015.07.010

[3] Bacchetta J , Fargue S , Boutroy S , et al. Bone metabolism in oxalosis: a single‐center study using new imaging techniques and biomarkers. Pediatr Nephrol. 2010;25(6):1081‐1089. 20213134 10.1007/s00467-010-1453-x

[4] Behnke B , Kemper MJ , Kruse HP , Muller‐Wiefel DE . Bone mineral density in children with primary hyperoxaluria type I. Nephrol Dial Transplant. 2001;16(11):2236‐2239. 11682674 10.1093/ndt/16.11.2236

[5] van der Hoeven SM , van Woerden CS , Groothoff JW . Primary hyperoxaluria type 1, a too often missed diagnosis and potentially treatable cause of end‐stage renal disease in adults: results of the Dutch cohort. Nephrol Dial Transplant. 2012;27(10):3855‐3862. 22844106 10.1093/ndt/gfs320

[6] Lieske JC , Monico CG , Holmes WS , et al. International registry for primary hyperoxaluria. Am J Nephrol. 2005;25(3):290‐296. 15961949 10.1159/000086360

[7] Garrelfs SF , Frishberg Y , Hulton SA , et al. Lumasiran, an RNAi therapeutic for primary hyperoxaluria type 1. N Engl J Med. 2021;384(13):1216‐1226. 33789010 10.1056/NEJMoa2021712

[8] Bia MJ , Insogna K . Treatment of sarcoidosis‐associated hypercalcemia with ketoconazole. Am J Kidney Dis. 1991;18(6):702‐705. 1962657 10.1016/s0272-6386(12)80613-5

